# Barriers of and strategies for shared decision‐making implementation in the care of metastatic breast cancer: A qualitative study among patients and healthcare professionals in an Asian country

**DOI:** 10.1111/hex.13590

**Published:** 2022-09-13

**Authors:** Ping Yein Lee, Ai Theng Cheong, Sazlina Shariff Ghazali, Aneesa Abdul Rashid, Siu Ching Ong, Soo Ying Ong, Adlinda Alip, McCarthy Sylvia, May Feng Chen, Nur Aishah Taib, Maheswari Jaganathan, Chirk Jenn Ng, Soo‐Hwang Teo

**Affiliations:** ^1^ UMeHealth Unit, Faculty of Medicine University of Malaya (UM) Kuala Lumpur Malaysia; ^2^ Department of Family Medicine, Faculty of Medicine and Health Sciences Universiti Putra Malaysia (UPM) Kuala Lumpur Malaysia; ^3^ Cancer Research Malaysia (CRMY) Subang Jaya Selangor Malaysia; ^4^ Department of Clinical Oncology, Faculty of Medicine University of Malaya (UM) Kuala Lumpur Malaysia; ^5^ Clinical Service Department Hospis Malaysia Kuala Lumpur Malaysia; ^6^ Department of Surgery, Universiti Malaya Cancer Research Institute, Faculty of Medicine University of Malaya (UM) Kuala Lumpur Malaysia; ^7^ Department of Primary Care Medicine, Faculty of Medicine University of Malaya (UM) Kuala Lumpur Malaysia; ^8^ Health Services & Systems Research Duke NUS Medical School Singapore Singapore; ^9^ SingHealth Polyclinics Singapore Singapore

**Keywords:** metastatic breast cancer, qualitative research, shared decision‐making

## Abstract

**Background:**

Shared decision‐making has been shown to improve the quality of life in metastatic breast cancer patients in high‐literacy and high‐resource settings. However, limited studies have examined the cultural preferences of metastatic breast cancer patients with shared decision‐making implementation and the barriers encountered in an Asian setting where societal norms predominate and physician decision‐making is at the forefront. This paper aims to identify (1) barriers to practising shared decision‐making faced by healthcare professionals and patients and (2) strategies for implementing shared decision‐making in the context of metastatic breast cancer management in Malaysia.

**Methods:**

We conducted a qualitative study involving 12 patients diagnosed with metastatic breast cancer, 16 healthcare professionals and 5 policymakers from surgical and oncology departments at public healthcare centres in Malaysia. Semi‐structured in‐depth interviews and focus group discussions were conducted. The interviews were recorded, transcribed verbatim and analysed using the thematic approach. Nvivo software was used to manage and analyse the data.

**Results:**

Five main themes emerged from the study: healthcare provider–patient communication, workforce availability, cultural and belief systems, goals of care and paternalism versus autonomy. Other strategies proposed to overcome barriers to implementing shared decision‐making were training of healthcare professionals and empowering nurses to manage patients' psychosocial issues.

**Conclusion:**

This study found that practising shared decision‐making in the public health sector remains challenging when managing patients with metastatic breast cancer. The utilization of decision‐making tools, patient empowerment and healthcare provider training may help address the system and healthcare provider–patient barriers identified in this study.

**Patient or Public Contribution:**

Patients were involved in the study design, recruitment and analysis.

## 1 INTRODUCTION

Shared decision‐making has improved quality of life and health outcomes in metastatic breast cancer patients in high‐literacy and high‐resource settings.^1^, ^2^, ^3^, ^4^, ^5^ Shared decision‐making allows patients and their clinicians to collaborate and come to a decision that incorporates patients' values and known evidence in the healthcare setting.^6^ Breast cancer patients in low‐resource settings often have delayed diagnosis with a late presentation; therefore, survival is significantly lower.^7^, ^8^, ^9^, ^10^ This may be due to the low literacy level of the patients in these settings, causing limited participation in the healthcare process.^11^, ^12^ Furthermore, because of the complexity of metastatic breast cancer and the heterogeneity in its treatment, patients may have limitations in comprehending the information.^13^ Shared decision‐making is therefore useful in providing optimal care to these patients.^13^

However, in an Asian setting where societal norms predominate, patients seemed to prefer physician‐ and family‐based decision‐making over shared or active decision‐making.^14^, ^15^ In a study of breast cancer patients in Malaysia, nearly half of the patients (42.6%) preferred passive decision‐making.^16^ This was compounded by the misperception of the physicians that more than half of these patients played an active role in decision‐making, highlighting a significant discordance between patients and physicians.^16^ The adoption of family‐centred decision‐making is the norm in Asia as patients perceive that family dynamics supersede their self‐care, but the negative consequences of this mode of decision‐making are often not explored.^17^, ^18^, ^19^ To date, limited studies have examined the cultural preferences of metastatic breast cancer patients with shared decision‐making implementation and the barriers encountered surrounding this.^12^ Therefore, it is unclear whether shared decision‐making has a similar impact on the quality of life in Asian patients.

This paper aims to explore (1) barriers to practising shared decision‐making faced by healthcare professionals and patients and (2) strategies for implementing shared decision‐making in the context of metastatic breast cancer management in a low‐resource Asian setting.

## 2 METHODS

### 2.1 Design

This was a qualitative study using focus group discussions (FGDs) and in‐depth interviews (IDIs) for patients, healthcare professionals and policymakers. The qualitative approach allows us to explore their perspectives on the barriers to the implementation of shared decision‐making in metastatic breast cancer treatment and strategies to improve its implementation. The women were interviewed one to one to create a safe space for them to voice their opinions. Healthcare professionals' accounts were garnered via FGDs to prompt discussions about the challenges they face in their healthcare experiences and IDIs for those who were unable to attend the FGDs. Policymakers' narratives were collected through IDIs.

### 2.2 Healthcare context

Malaysia operates a two‐tiered healthcare system (public and private). The public healthcare sector is heavily funded by government taxes and run by the Ministry of Health. In public hospitals, patients are charged MYR5 (USD1.20) per specialist clinic consultation. Public hospitals cover both urban and rural areas. On the other hand, the private healthcare sector requires patients to pay out of pocket and is normally centred in urban areas. There are also many nongovernmental organizations (NGOs) that offer various health services such as patient navigation and palliative care. The usage of traditional medicine among Malaysian patients, especially the Chinese, Malay and Indian populations, is prevalent due to the local cultural beliefs. Many healthcare professionals in the public and private sectors play a role in metastatic breast cancer care.^20^

### 2.3 Study setting

The study was conducted in Malaysia at the following locations: one government district hospital (Hospital Tengku Ampuan Rahimah, Klang), one academic institution (Universiti Malaya Medical Centre), three NGOs (Cancer Research Malaysia, Hospis Malaysia, National Cancer Society Malaysia) and one patient support group (Breast Cancer Welfare Association). Study participants were recruited from these different sites to maximize the diversity of participants from different ethnic and social–economic backgrounds and different healthcare settings.

### 2.4 Research team and reflexivity

The research team was led by a family medicine specialist (L. P. Y.) with a special interest in shared decision‐making research and with prior experience in leading qualitative research.^21^ The study design was developed by the principal investigator with four family medicine specialists who have experience in shared decision‐making and qualitative research (N. C. J., C. A. T., S. S. G., A. A. R.): one breast surgeon (N. A. T.), one clinical oncologist (A. A.), one oncology medical officer (C. M. F.), one researcher with expertize in breast cancer research (T. S. H.) and two medical graduates (O. S. C. and O. S. Y.). All FGDs and IDIs were conducted by four research team members (L. P. Y., C. A. T., S. S. G., A. A. R.) who have 7–15 years of experience with qualitative evaluation and focus group facilitation. All members of the research team, with the exception of N. C. J., were female.

### 2.5 Participants, recruitment and sampling

#### 2.5.1 Recruitment of patients, healthcare professionals and policymakers

We used purposive sampling to identify female patients aged at least 18 years who were newly diagnosed with metastatic breast cancer as well as healthcare professionals and policymakers who had been continuously involved in the management of metastatic breast cancer patients for the past 6 months. Purposive sampling was carried out to maximize the diversity of participants from different ethnicities, social–economic backgrounds and healthcare settings. Participants were approached face to face at the outpatient breast clinics during their routine follow‐up or their offices (for healthcare professionals and policymakers). If participants consented, individual IDIs were arranged for patients (to ensure discussion of sensitive issues and emotional support to the participants) and policymakers (for logistic reasons), whereas FGDs or IDIs (if time constraints existed) were arranged for healthcare professionals. To ensure homogeneity and to capitalize on shared experiences among the healthcare professionals,^22^ FGDs were arranged to include healthcare professionals from similar practice backgrounds and locations.

The interviews were conducted sequentially with initial interviews with healthcare professionals, followed by interviews with policymakers and finally interviews with patients. This allowed the researchers to analyse and capture the main challenges and issues experienced by healthcare professionals and guide the subsequent interviews of the policymakers and patients.

Written informed consent was obtained for all participants. Participants received a small financial acknowledgement (RM100 [USD24] to RM150 [USD36]) on completion of interviews to help offset travel and other costs incurred.

The sample size of this qualitative study was determined by data saturation.^23^ Recruitment was stopped after 25 IDIs (12 patients, 8 healthcare professionals and 5 policymakers) and 3 healthcare professional FGDs, when researchers agreed that the analysis had reached thematic saturation.

### 2.6 Data collection

We developed a semi‐structured interview guide by adapting the topic guide utilized in a previous qualitative study by Adina et al. in 2013 that incorporated the Ottawa Decision Support Framework and further modified and improvised through literature reviews and expert opinions.^13^, ^24^, ^25^, ^26^ The topic guide (Supporting Information: Appendices S6 and S7) covered the barriers in decision‐making, decision‐making experiences and information needs of patients; healthcare professionals' or policymakers' challenges in the implementation of shared decision‐making; and their experiences with metastatic breast cancer patients. The interview guide was preliminarily tested among five metastatic breast cancer patients and modified to improve its clarity. Open‐ended questions were used to facilitate open discussion and prompts were introduced when the interview did not evoke important issues that should be covered. Interviews were conducted from March to August 2019. All interviews lasted approximately 60 min and were conducted by L. P. Y., C. A. T., S. S. G. or A. A. R., with an assistant present detailing field notes on verbal and nonverbal cues. No repeat interviews were carried out. The interviews were audio‐recorded and transcribed verbatim. An independent transcriber checked the transcripts for accuracy. The checked transcript was then used for data analysis. The interviews and data analysis were conducted concurrently.

### 2.7 Data analysis and validation

We used NVivo 12 software to manage the qualitative data. The transcripts were analysed inductively using the thematic approach, which included the following steps: familiarization; identifying themes; indexing; charting; and mapping.^27^ Two researchers (S. S. G. and A. A. R.) analysed the patients' data, whereas two other researchers (L. P. Y. and C. A. T.) analysed the healthcare professionals' and policymakers' data separately. All the analyses were performed independently. A list of free nodes (codes) was created and subsequently, all the potentially relevant codes were extracted into themes. The themes were then merged to form categories and a coding framework. Any disagreements that arose were discussed during research meetings and were resolved via a final consensus on common emerging themes obtained among all researchers. Participants were asked to provide feedback on the findings, and there was no disagreement with the analysis.

### 2.8 Ethics approval

This study received ethical approval from the Medical Research and Ethics Committee of the Ministry of Health, Malaysia (NMRR ID: NMRR‐18‐929‐44182). All participants provided written informed consent.

## 3 RESULTS

### 3.1 Description of the participants

A total of 33 participants were included in the study: 12 patients, 16 healthcare professionals (3 clinical oncology nurses, 3 breast care nurses, 1 palliative care nurse, 3 surgical medical officers, 2 palliative care physicians, 1 clinical oncologist, 1 clinical oncology trainee, 1 surgical consultant, 1 patient navigation programme coordinator and 1 physiotherapist) and 5 policymakers consented to participate in the study. Two patients declined due to time constraints. The median age of the patients was 53 years (interquartile range [IQR] = 15, range = 34–69), and the mean age at breast cancer diagnosis was 45 years. Healthcare professionals were working in university hospitals, public government hospitals and NGOs, with a median working experience of 17 years (IQR = 10, range = 3.5–27). Policymakers had a median age of 53 years (IQR = 15.5, range = 40–57) and had working experience ranging between 9 and 32 years, with a median of 20 years (IQR = 15.5, range = 9–32). Tables A1 and A2 outline the sociodemographic data of the participants.

### 3.2 Barriers and strategies for shared decision‐making implementation

Five themes emerged on the barriers and strategies identified for shared decision‐making: workforce availability; healthcare provider–patient communication; cultural and belief systems; goals of care; and paternalism versus autonomy. Tables A3, A4, A5 summarize the individual barriers, similar barriers and suggested strategies among the participants.

### 3.3 Workforce availability

#### 3.3.1 Barriers

All participants agreed that time constraint (due to limited workforce) is a significant challenge in allowing for a proper explanation, counselling and decision‐making. Patients felt that they could not ask the doctors questions due to the perception that the doctors were busy and had time constraints. In terms of treatment decision‐making, the majority of the healthcare professionals provided patients with 1–2 weeks to make decisions, but patients sometimes took longer to decide, as their concerns were not being addressed due to time constraints. One healthcare professional thought it was reasonable to provide patients with more time to gather information and make important decisions but was peer‐pressured by colleagues and the short time allocated by the system. In addition, the lack of continuity of care (patients see different doctors at each consultation in public hospitals) had contributed to a lack of individualized discussion of patients' concerns. Healthcare professionals and policymakers felt that these system barriers were due to the lack of resources, human power and expertize (31 oncologists across 6 oncology centres throughout the country) and the lack of a robust patient support system, resulting in certain healthcare professionals like nurses taking on multiple roles (e.g., social worker, counsellor).

*Yes, actually I wanted to know [about my condition or treatment]. But there's always not enough time*. P07, 34 years old, diagnosed at 30 years old

*…Usually, we hope that they can decide on the same visit, but I think it's quite selfish. Sometimes your colleague will say, ‘How come you don't ask the patient to make a decision now? Instead, you ask the patient to come back two weeks after [later]?’ … Yeah, so usually I will usually ask patients to make decisions on the spot because we don't want others to think that you are not doing your job*. HCP005, healthcare professional, university hospital, 3.5 years of working experience

*Hospital B, they always change doctors. Whatever the first doctor said [in the previous consultation], and [whatever] the second doctor [says in the next consultation is] very different*. P05, 59 years old, diagnosed at 52 years old

#### 3.3.2 Strategies

Involving other parties, such as patient support groups and NGOs, to provide support and counselling for patients and the availability of social welfare support for financial assistance may pave the way to set‐up of a robust system to support patients in the future. The lack of continuity of care has been suggested to be resolved by assigning a patient navigator to each patient. Healthcare professionals can involve patient navigators in patients' treatment decision‐making to provide support and allow patients to clear their doubts outside of consultations, as a solution to the time constraint issues. Policymakers noted that access to expensive innovative therapies remains a challenge in low‐resource settings and this added to the decisional conflict for patients. In this context, they highlighted the need for a centralized policy on value‐based medicine so that the healthcare system could put in place funding for innovative medicines that can prolong or improve the quality of life.

*…We must also look at value‐based medicine. Although the treatment may be efficacious, … but at the same time we need to look at the whole population…*. PM03, policymaker, government hospital, 9 years of working experience

### 3.4 Healthcare provider–patient communication

#### 3.4.1 Barriers

Patients felt that their personal values or concerns were often not addressed and they received no proper explanations from the healthcare professionals. This is made worse by the complexity of the oncological treatments in metastatic breast cancer. Their questions were often met with dismissive remarks from the healthcare professionals. Patients also desired explanation and discussion regarding the side effects and felt that the healthcare professionals avoided talking about this on purpose. On top of this, they faced judgemental attitudes from the healthcare professionals when seeking care, causing them to avoid hospital‐based treatment and turn to alternative treatments. However, healthcare professionals were unfamiliar with alternative treatments and found it difficult to advise patients on their use. These challenges raised by patients were consistent with what healthcare professionals and policymakers have observed. Healthcare professionals also reported that language barriers (especially in a multilingual country) and patients being in denial or not listening to the doctors and missing out on information impacted the quality of patient education and counselling.

*That doctor in Hospital A, I think the explanation [about treatment] is not clear, she always [dismisses my question]*. P04, 39 years old, diagnosed at 35 years old

*…there are some[patients] that knows [about the side effects] but a lot of them, they do not know… Some [of them will] ask. Some, they don't ask. Because sometimes they're scared that the doctor will scold them or [there is] no time for the explanation*. HCP009, healthcare professional, NGO, 21 years of working experience

*Some specialists scold the patients who use alternative medicine…*. PM03, policymaker, government hospital, 9 years of working experience

#### 3.4.2 Strategies

The use of booklets was suggested to tackle the complexity of oncological information, which will in turn address patients' concerns and provide adequate information. Patients also conveyed the need for a counsellor for emotional and information support upon receiving the diagnosis. Healthcare professionals and policymakers suggested organizing training for healthcare professionals on educating and counselling patients and respecting patients' treatment decisions while supporting them when they decide to return to their medical treatment from alternative treatment. This can promote nonjudgemental attitudes in patient management. Policymakers also suggested increasing the authority given to nurses towards assisting with psychosocial issue management to properly address patients' concerns and requiring patients to bring someone along for consultation to retain the information given.

*…The internet will do. Or like, if, it would be nice also if there's this one nice booklet you can give. Ok, you've been diagnosed, this is what you should know. Then you can read it at your own time, right?* P02, 34 years old, diagnosed at 33 years old

*Nurses [are] better [at communication and counselling]. But they are not given enough authority. … If they disclose information, the doctors will scold them*. PM01, policymaker, NGO, 32 years of working experience

### 3.5 Cultural and belief systems

#### 3.5.1 Barriers

Patients and healthcare professionals have a misconception that palliative care is only for those who are at the end stage of thier cancer and therefore shy away from it. Most patients also rely on their husbands or their families to make the decisions. In the Malay and Indian communities, healthcare professionals reported that most patients looked to their husbands to make the decision, especially regarding mastectomy, as society often perceives that women should have breasts and husbands are considered to have more authority in their religious and cultural settings. Healthcare professionals also believed that all ethnicities were prone to using alternative treatments as these were believed to cure cancer and that these treatments were more ‘natural’ and less harmful to the body compared to Western medicine. One policymaker noted that Asians were less receptive to support groups due to the lack of openness as compared to their Western counterparts, making it a less suitable patient support method for Asians.

*I try [not] to know about [palliative care]… I know [it] is for very end stage cancer patient… I think “Eh, I still don't need it now*. P03, 47 years old, diagnosed at 45 years old

*I depend on my family for all my decisions, actually…*. P07, 34 years old, diagnosed at 30 years old

*For me [my observation], the husband has the most [influence]… Because it's the breast… This is a spiritual and cultural thing for our society. This influence is most prominent in the Malay and Indian communities*. HCP001, healthcare professional, university hospital, 9–25 years of working experience

#### 3.5.2 Strategies

Patients considered family and religious support important, and this is interlinked to the healthcare professionals' and policymakers' suggestion to bring out the patient's voice among family members. As important as family support is, healthcare professionals and policymakers think that the patient's decision should still be prioritized. As for the lack of knowledge of healthcare professionals regarding alternative treatments, policymakers suggested training for healthcare professionals in this field and patients also suggested that alternative medicine expertize be made available. Healthcare professionals and policymakers also noted that patients become receptive to palliative care once the misconceptions are cleared.

*…I would want to … bring out the patient's voice … where other families dominate the conversation*. HCP002, healthcare professional, NGO, 19 years of working experience

*It's the whole idea to say it's hospice is not there for you to die‐… Hospice is there for you to have a good quality of life, to help you live*. PM02, policymaker, NGO, 20 years of working experience

### 3.6 Goals of care

#### 3.6.1 Barriers

There was a mismatch of treatment goals between patients, their family members and healthcare professionals. Patients' treatment goals are often centred on their life goals, but healthcare professionals focus on health outcomes. In addition, patients have an expectation of a cure for their metastatic disease but healthcare professionals, based on the knowledge that there is no cure for the disease, aimed to prolong patients' lives. Hence, patients felt that the prognosis of their disease was often not discussed by their treating doctors. It was considered to be an important piece of information for them at the time of diagnosis. However, retrospectively, some patients no longer think it is necessary. Doctors also felt that they were not well equipped to decide on when to stop treatment.

*No, they did not talk about the prognosis… On one hand, I would like to know what to expect. But, on the other hand, maybe it's better [if] I don't know. … At first, I was quite upset that she didn't talk to me about it (how long is my survival)… but… [in] hindsight, maybe it's a good thing also she didn't. Because… I survived longer than what was stated in the internet*. P02, 34 years old, diagnosed at 33 years old

#### 3.6.2 Strategies

Therefore, patients' goals of treatment have to be explored instead of just focusing on their health outcomes. The fact of the incurable nature of the disease should also be conveyed honestly to the patients to enable them to have realistic expectations of their disease and for decision‐making.

*We should avoid giving them something (information that may not be true) that we think is hopeful for them, but it is actually not*. HCP010, healthcare professional, government hospital, 19 years of working experience

### 3.7 Paternalism versus autonomy

#### 3.7.1 Barriers

All groups identified that Asian patients are accustomed to being at the receiving end of the decision‐making process and are often confused or surprised when the decision‐making role is handed over to them. When told to recall whether treatment options were discussed at the time of diagnosis and decision‐making, some patients and healthcare professionals recalled doctors adopting a paternalistic approach and directly starting the patients on treatment.

*[The doctor] asked me to decide (my treatment), I said, ‘Why [do] you let me decide’, I'm not sure how is it, I don't know. So you[‘re supposed to’] recommend which one you think is the best*. P03, 47 years old, diagnosed at 45 years old

For some families where the patient is not the key decision‐maker, family members were informed of the diagnosis before the patient and they made decisions on behalf of the patient. Policymakers pointed out that in certain cultural settings, spousal consent is required for all matters that are related to reproductive issues including treatment regarding breast and other cancers in women.

*I think [the doctors] weren't expecting cancer. But… They broke the news to my parents and my husband first*. P02, 34 years old, diagnosed at 33 years old

*…We have the ridiculous …spousal consent. And some doctors will say the married woman doesn't have a right to a decision*. PM01, policymaker, NGO, 32 years of working experience

#### 3.7.2 Strategies

Some patients have recognized the importance of playing an active role in asking the healthcare professionals any doubts they may have. However, all patients should be empowered and educated on their decision‐making roles, as suggested by one policymaker. Healthcare professionals suggested tackling the ethical issues by first determining the degree of family involvement patients want and to involve patients' family members in the decision‐making process for support, but not to make decisions for the patients.

*I think for patients, we have to actually ask the doctor. Doctors won't know actually what information you need*. P03, 47 years old, diagnosed at 45 years old

*…some patients want decisions to be made for them, some people want to be shared, some wants to make it on their own. …Work with the patient. Know what is their way of making decisions*. PM01, policymaker, NGO, 32 years of working experience

## 4 DISCUSSION

In this paper, we demonstrated that there are many barriers and corresponding strategies in implementing shared decision‐making in metastatic breast cancer management. Five main themes emerged from the thematic analysis: (1) healthcare provider–patient communication, (2) workforce availability, (3) cultural and belief systems, (4) goals of care and (5) paternalism versus autonomy. A few noteworthy findings were obtained.

Our findings corroborate those of previous studies that have reported decisional conflicts and discrepancies in treatment goals in healthcare provider–patient–caregiver communication.^28^, ^29^, ^30^, ^31^, ^32^ There also exists a discordance in patients' healthcare needs assessment between healthcare providers and patients, whereby patients expected encouragement and spiritual support, but healthcare providers focused on treatment efficacy.^33^ As one participant mentioned, the training of healthcare providers focuses on healthcare outcomes and not the patient's life goals. Healthcare providers may also believe that they understand the patients' preferences and therefore neglect what patients express.^34^, ^35^ Healthcare professionals usually inform the patients about the possible side effects of the treatment, and warn them that there are no guarantees that the treatment will work for them individually. However, alternative medicine at times promises a cure with no side effects, which is preferred by many patients. Patients may also turn to alternative treatments if their concerns regarding Western medicine are not properly addressed or if their treatment expectations are not in line with the promises or actual outcomes.^36^, ^37^, ^38^

Our study also found barriers that are pertinent to our setting—a multiracial and multilingual Southeast Asian country. Most studies on consent and decision‐making in breast cancer treatments among couples have reported on joint decision‐making.^39^, ^40^, ^41^ Obtaining spousal consent from husband for all matters related to reproductive issues were practiced by some healthcare proffessionals in certain cultural settings.^42^ Due to cultural expectations and obligations, many Asian women depend on their husbands or sons fully to make these decisions as the ‘head of the household’.^43^, ^44^ The practice of spousal consent suggested in our study contradicts the dyadic approach that is adopted by most couples in breast cancer treatment decision‐making.^39^, ^40^, ^41^ As breast cancer affects not only the patients but also their families, it is important for healthcare professionals to involve the partner in decision‐making, but promote a joint decision‐making approach.^41^, ^45^, ^46^, ^47^

Previous studies have demonstrated that there are discrepancies between patients' preferred and actual roles in making cancer treatment decisions.^16^, ^48^, ^49^ Our study found that our patients were not aware that their participation in decision‐making is allowed, indicating that they are unclear about different decision‐making roles in breast cancer treatment. Only one study on patients with pulmonary nodules found that some patients were unaware of their role in decision‐making or that a decision was being made in their actual roles.^50^ Many patients also did not expect to be involved in treatment decision‐making and this fuels the paternalistic approach of healthcare professionals who assume that they know the patients' preference, therefore leaving patients out of the picture during decision‐making.^34^, ^35^ The paternalistic approach that healthcare professionals utilized might also be due to their lack of belief in shared decision‐making as one of the challenges, as highlighted in the review by Triantaphyllou et al.^51^ This finding adds to what is already known about treatment decision‐making in the oncology field. Patients should also be educated and empowered to ask questions and participate in decision‐making, which could be made possible via workshops utilizing role‐playing and communication exercises.^52^ As suggested by Triantaphyllou et al.,^51^ new laws and policies for shared decision‐making have also been proposed to be enacted and enforced in the country when our population is ready for it.^42^

We found that most barriers to shared decision‐making in metastatic breast cancer care are similar to what is reported in other studies regarding shared decision‐making. These include time constraints, challenges in communication and treatment decision uncertainties, which appear to be the most important considerations regardless of the setting.^51^, ^53^, ^54^, ^55^ Similar to the challenges highlighted in a review, ^51^ our patients have expressed difficulties in understanding the treatment selection process due to the complex nature of oncological treatments. A possible explanation is that the healthcare providers have to follow the timing for consultations that are set by the schedule, as well as the requirement to complete other clinical and administrative tasks, resulting in them hurrying explanations and providing patients with considerable complex medical information.^56^ Healthcare providers usually utilize lengthy monologs and medical jargon to speed up the consultation and fail to explore patients' preferences and confirm patients' understanding, causing communication challenges and leading to treatment decision uncertainties.^53^, ^57^ The suggestions to include oncology‐trained nurses for patient education and task reallocation to improve communication and curb time issues may be considered.^57^ In addition, the use of decision aids (a visualized tool) may help to improve patients' understanding of the complex treatment regime for a better shared decision‐making process and communication with the healthcare providers.^51^, ^58^ The findings of this study have guided the researchers in the development of patients' decision aids in metastatic breast cancer.^59^

Some barriers that we identified were also found in other studies but not in those that are predominantly in European populations. These were the strong family involvement in their medical decision‐making, language barriers and paternalism in healthcare.^54^, ^55^ Asian patients are largely dependent on their families to make decisions and family involvement often raises concerns about patient autonomy, as there was collusion involved.^60^, ^61^ The degree of family involvement is influenced by cultural obligations and beliefs; therefore, family members perceive the need to be involved in the disease management.^62^ However, family involvement may also be a positive influence in supporting the patient and promoting the patient's autonomy and hence shared decision‐making, as patients also take into account their family life in treatment decision‐making.^63^ The language barrier exists in multiethnic and multilingual countries, or in instances where foreigners reside in other countries.^64^, ^65^ Traditionally, the Asian model of healthcare adopts a paternalistic approach and patients generally trust the physicians to make the decisions, in turn contributing to patients being unaware of their decision‐making role.^66^, ^67^

As this study included patients from diverse background and age group, healthcare professionals and policymakers from diverse setting and levels of experiences, it captures the spectrum of barriers that exist in our setting. The qualitative study design allowed an in‐depth exploration of the barriers that hindered the process of shared decision‐making among patients and healthcare professionals. Our study is the first to look into how cultural influences hinder shared decision‐making in metastatic breast cancer care in a Southeast Asian setting and provide strategies to resolve these. We also garnered policymakers' views to understand the big picture of metastatic breast cancer management from a public health perspective and suggest appropriate strategies to curb system barriers.

We may be able to apply these data to implement suggested strategies in metastatic breast cancer patients and other types of life‐critical diseases for similar populations. However, we acknowledge that our study has several limitations. Patients were mainly from the Chinese ethnic group and therefore our findings may not be as relevant to other patient groups. In addition, as the study was conducted in urban and semi‐rural areas, the findings may not be transferable to people from rural settings.

## 5 CONCLUSIONS

This study found that practising shared decision‐making in the public health sector remains challenging when managing patients with metastatic breast cancer. The utilization of decision‐making tools, patient empowerment and healthcare provider training may help address the system and healthcare provider–patient barriers identified in this study but may not be able to address the chronic shortage in oncology healthcare providers in these settings. In addition, the findings of this study are from a Southeast Asian country (Malaysia) and may not be generalizable to other settings.

## AUTHOR CONTRIBUTIONS

Ping Yein Lee, Ai Theng Cheong, Sazlina Shariff Ghazali, Aneesa Abdul Rashid, Soo Ying Ong and Siu Ching Ong were involved in data collection, analysis and writing of the original draft of the manuscript. All authors contributed to the funding acquisition, conceptualization, writing, editing and review of the manuscript. All authors approved the final version.

## CONFLICT OF INTEREST

The authors declare no conflict of interest.

## Supporting information

Supporting information.

Supporting information.

## Data Availability

The data that support the findings of this study are available from the corresponding author upon reasonable request. The data are not publicly available as they include information that could compromise research participant privacy.

## A

### 1

See Tables A1, A2, A3, A4, A5.

**Table A1:** Sociodemographic information of healthcare professionals and policymakers

Demographics		Healthcare professionals (*N* = 16)	Percentage (%)	Policymakers^a^ (*N* = 5)	Percentage (%)
Age					
30–39 years old		7	43.75	0	0
40–49 years old		8	50	2	40
50–59 years old		1	6.25	3	60
Gender					
Male		1	6.25	3	60
Female		15	93.75	2	40
Race					
Malay		11	68.75	2	40
Chinese		4	25	1	20
Indian		1	6.25	1	20
Current position					
Nurses		7	43.75	0	0
Medical officer/trainee		2	18.75	0	0
Clinical specialists/consultant		4	25	0	0
Others		2	12.5	5	100
Patient supportive care healthcare professionals		2	12.50	0	0
NGO, public and private setting policymakers		0	0	5	100
Site of practice					
Government hospital		4	25	3	60
University hospital		10	62.5	0	0
Others		2	12.5	2	40
NGO		2	12.5	2	40
Years of working experience					
<10		3	18.75	1	20
10–19		8	50	1	20
20–29		5	31.25	2	40
30–39		0	0	1	20

Abbreviation: NGO, nongovernmental organization.

^a^ One policymaker declined to report race.

**Table A2:** Similar barriers to shared decision‐making implementation identified by all groups of participants

Themes	Patients	Healthcare professionals/policymakers
Healthcare provider–patient communication	Patients' personal values, concerns and misconceptions not explored. Judgemental attitudes of healthcare professionals. Difficulties in advising patients who use alternative medicine. Patients face difficulty understanding complex treatment options. No proper explanation regarding treatment and its side effects. Patients in denial.	
Workforce availability	Time constraints in consultations and decision‐making. Lack of continuity of care due to different doctors seen at each consultation.	
Cultural and belief systems	Stigma and misconceptions towards palliative care. Dependence on and influence of others in decision‐making.	
Goals of care	Discordance in treatment goals between patients and healthcare professionals (e.g., patients expect cure or support; healthcare professionals focus the discussion on efficacy).	
Paternalism versus autonomy	Treatment options were not discussed by doctors. Patients are unaware that they can play an active role in decision‐making.	

**Table A3:** Individual barriers to shared decision‐making implementation by groups

Themes	Patients	Healthcare professionals	Policymakers
Healthcare provider–patient communication		Missing out information and language barriers during counselling. Patients refuse to listen to the specialists.	Patients seek multiple opinions, leading to decision‐making delay.
Workforce availability		Lack of manpower or expertize. No robust system for patients to get support.	Medical professionals face time constraints due to administrative tasks. Lack of resources.
Cultural and belief systems	Role as a mother and breadwinner.	Societal expectation on women to have breasts. Patient's beliefs regarding alternative treatment (e.g., as cure or immune system booster).	Differences in Asian and Western cultures lead to different reception to support groups. Lack of manpower or expertize. No robust system for patients to get support.
Goals of care	Patients sought prognosis information, but was not discussed.	Treatment cessation matters (i.e., when to stop treatment).	
Paternalism versus autonomy	Breaking of bad news not done for patients first.	Collusion by family members.	Collusion by family members. The practice of spousal consent.

**Table A4:** Suggested strategies to curb shared decision‐making implementation issues

Themes	Suggestions
Healthcare provider–patient communication	Use certain educational resources to tackle the complexity of oncological treatment information. Clear explanation on treatment decisions needed from healthcare professionals. Organize trainings for healthcare professionals on educating and counselling patients. Healthcare professionals should respect patients' treatment decision and support them when they decide to return to the healthcare scene. Counsellor should be provided to patients for support after diagnosis. Healthcare professionals should have nonjudgemental attitudes. More authority should be given to nurses to assist in psychosocial issue management. Patients are advised to bring someone along during consultations to tackle patient forgetfulness.
Workforce availability	Other parties (e.g., NGOs, patient support groups) can be involved in supporting and counselling patients. Ensure continuity of care by providing a fixed healthcare provider for the patients. Allow time for patients to make decisions with the facilitation of patient navigators. Practice of value‐based medicine.
Cultural and belief systems	Involve family members in decision‐making to avoid conflict. Healthcare professionals should being out the patient's voice among family members. Family and religious support should still be allowed. Misconceptions about palliative care should be addressed. Alternative medicine expertize should be provided to patients (e.g., referring to the Chinese medicine department in other hospitals). Training of healthcare professionals in alternative medicine should be provided.
Goals of care	Healthcare professionals should explore the patient's life goals and not just health outcomes. Honesty and transparency in communication regarding the incurable nature of the disease should be ensured to the patients.
Paternalism versus autonomy	Patients should play an active role in asking the healthcare professionals questions. Healthcare professionals should work with patients to identify decision‐making roles. Healthcare professionals should address confidentiality and ethical issues with sensitivity. Healthcare professionals should involve the patients' family members in the decision‐making process for support but not to make decisions for the patients.

Abbreviation: NGO, nongovernmental organization.

**Table A5:** Sociodemographic information of patients

Demographics		Patients (*N* = 12)	Percentage (%)
Age			
30–39 years old		3	25
40–49 years old		2	16.75
50–59 years old		6	50
60–69 years old		1	8.25
Gender			
Female		12	100
Race			
Malay		4	33.3
Chinese		7	58.3
Indian		1	8.3
Marital status			
Single		2	16.75
Married		9	75
Divorced		1	8.25
Highest education level			
Secondary school		4	33.25
Diploma		2	16.75
University		6	50
Current occupation			
Unemployed/homemaker		5	41.75
Government servant		3	25
Private sector		1	8.25
Retired		2	16.75
Others		1	8.25
Total monthly household income			
≤RM1000		1	8.25
RM1001–RM5000		6	50
RM5000–RM10,000		2	1.75
≥RM10,000		3	25
Source of financial support			
Self‐funding		4	33.3
Savings		1	8.3
Insurance		3	25
Others		4	33.3
Age of diagnosis			
30–39 years old		3	25
40–49 years old		4	33
50–59 years old		5	42
Comorbidities			
Yes		3	25
No		9	75
