# Supplementary material for: Barriers of and strategies for shared decision‐making implementation in the care of metastatic breast cancer: A qualitative study among patients and healthcare professionals in an Asian country
Source: Health Expect. 2022 Sep 13;25(6):2837–50. doi: 10.1111/hex.13590 (PMC9700188; doi:10.1111/hex.13590)
Supplement: Supplementary file 1 — Supporting information. [file HEX-25--s002.docx]

**Appendix 6: Topic guide for healthcare professionals and policy makers**

***Healthcare Professional Interview Topic Guide (Phase 1)***

**Preamble**:

- Ice-breaking
- Explain no right or wrong answer
- Need to get consent for the interview and recording
- Do not have to answer if the participant doesn’t wish to do so
- Explain that this interview is about women with early breast cancer

**Interview:**

***Role and Background***

1. What is your role in caring for women with breast cancer?

***Managing newly diagnosed patients***

1. Do you face any challenges when managing women with metastatic breast cancer?

- If yes, what are the challenges you face?
- Prompt: Keeping up with knowledge, communicating with patients, emotional effects
- What is needed to prepare you in facing these challenges?
  - Prompt: training, previous experience
- If no, why not?
- How do you address their concerns related to the diagnosis?
- What is the difference between managing women with early and late breast cancer?

***Patient’s experience***

1. Patients react differently to the diagnosis of breast cancer. Could you please share with us how your patients react to the diagnosis of metastatic breast cancer?

- How do they respond emotionally to the diagnosis?
- What are the questions they ask?
- What do they want to know?

***Patients’ decision making***

1. How does your patient make decisions?

- Timing – when? Describe the follow up process
- Based on information – where do they get the information
- Influence from others – who influences their decision
- Financial status

1. From your experience, who is involved in making decisions about the cancer treatment?

- Husband? Immediate family? Relatives? Friends? Self?

1. What is your role in helping patients to make decisions about their treatment?

- Prompts: Doctor makes the decision? Let the patient and family make the decision? Shared decision?

1. What are the treatment options for women with metastatic breast cancer?

Prompts: Surgical treatment etc.

1. Have you encountered women with metastatic breast cancer who refuse treatment?

- If yes, why did they refuse treatment?

***Helping patients to make decisions***

1. How do you help women with metastatic breast cancer to make decisions about their treatment?

- What do you do? What do you say?
- Do you explain the benefits of treatment to the patient?
  - If yes, how do you do that? (Prompt: Discuss in general? Use pamphlets? Draws?)
  - If no, why not?
- Do you explain the risks of treatment to the patient?
  - If yes, how do you do that? If no, why not?
- Do you explain the disease outcomes with and without treatment?
- Do you discuss the impact of the diagnosis on their work/daily lives/relationships?
- Do you discuss the feasibility of having the treatment, is. Social support, financial costs, days off work, duration of treatment?

1. Do you refer women with metastatic breast cancer to other healthcare professionals?

- If yes, who do you refer to?
- What is their role?
- Who are the patients that you refer?

1. What is your view about patients seeking alternative therapy for metastatic breast cancer?

- Do you discuss alternative therapy with your patients?
- What is your response when they raise the issue of alternative therapy?
- What are some of the alternative therapies patients often use?

***Support in managing breast cancer patients***

1. Counselling breast cancer patients can be difficult. Do you need any support when counseling patients with metastatic breast cancer about their treatment?

- If yes, what support do you need?
  - Probe: more time; manpower; information; training etc.

1. When helping patients to make treatment decisions, what is your view on (including the pros and cons):

- One-to-one counseling
- Peer support group
- Providing health information
- Use of patient decision aid

1. We are thinking of developing a decision aid for patients with metastatic breast cancer. (Explain about Patient Decision Aid) Do you think it would be useful for you and your patients?

- If no, why not?
- If yes:
  - What format? Prompt: paper, audio, CD, web-based
  - Who will benefit from it?
  - How would you use it? Probe: patient read by herself? With nurse? With doctor? With family?

1. Is there anyone (healthcare professionals/NGOs) whom you think we should talk to regarding this research project?

**Thank you**
