# Supplementary material for: Barriers of and strategies for shared decision‐making implementation in the care of metastatic breast cancer: A qualitative study among patients and healthcare professionals in an Asian country
Source: Health Expect. 2022 Sep 13;25(6):2837–50. doi: 10.1111/hex.13590 (PMC9700188; doi:10.1111/hex.13590)
Supplement: Supplementary file 2 — Supporting information. [file HEX-25--s001.docx]

**Appendix 7: Topic guide for patients**

***Patient Interview Topic Guide (Phase 1)***

**Preamble**:

- Ice-breaking
- Explain no right or wrong answer
- Need to get consent for the interview and recording
- Do not have to answer if the participant doesn’t wish to do so

**Interview:**

1. **When were you diagnosed with metastatic breast cancer?**

- How were you diagnosed?
- What did you know about your diagnosis?

1. **Who has informed you about the diagnosis?**

- How did you feel when you were told about your diagnosis?
- How did the doctor tell you about the diagnosis? How was the expression of your doctor?
- Did you remember what the doctor say?
- What did you want to know at that time? How your doctor help to address your concerns?

1. **What did the doctor/nurse say about the treatment?**

- Prompt: surgery (mastectomy, breast conservation surgery, chemotherapy, radiotherapy, immunotherapy, targeted therapy, palliative)
- What treatments did the oncologist recommend?

**Prompt: Was there any options given to you, where you had more than one choice of treatments? What were they?

- What do you know about the treatment options?
  - What do you think about surgery?
    - What are your concerns?
  - What do you think about chemotherapy?
    - What are your concerns?
  - What do you think about radiotherapy?
    - What are your concerns?
  - What do you think about immunotherapy?
    - What are your concerns?
  - What do you think about targeted therapy?
    - What are your concerns?
  - What do you think about palliative?
    - What are your concerns?
  - Did the doctor/nurse ask you what you want to know about the treatment?
- What do you think about alternative medicine?
  - What alternative medicine do you know?
    - Prompt: herbs, special diet therapy, spiritual healing, etc.
  - Where did you hear about alternative medicine?
  - Do you want to seek alternative treatment? If yes, why?
  - What are your concerns about alternative medicine?

1. **At this moment, what is your preferred treatment option?**

- Prompt: surgery, chemotherapy, radiotherapy, immunotherapy, palliative, alternative treatment
- Why?
- Why not the other treatment options?
- Is it difficult for you to make the decision?
  - If yes, why?
  - If not, why not?

1. **What will help you to (How do you) make a decision about the treatment of your breast cancer?**

- Who is involved in making the decision?
  - Prompt: Yourself only? Your family (who)? Your doctor? Others?
- Do you need more time?
- How do you cope?
- What do you need now?
  - More information?
  - More support from family?
  - More support from healthcare providers?
- Who should make the decision about your treatment?
  - Why?
- Prompt: Cost of treatments/ ability to get reimbursement/ financial support

1. **What information have you received so far?**

- Where did you receive this information?
- Who gave it to you?
- Do you find the information helpful?
- In what format was the information given? (Pamphlet? Verbal? Video?)

1. **What information do you want?**

- Prompt:
  - About metastatic breast cancer? (Give examples)
  - About the treatment options? (e.g. surgery, chemotherapy, radiotherapy, immunotherapy, targeted therapy, palliative)
  - About alternative medicine?
  - Others?
  - Cost
- How should the information be given?
  - Booklets?
  - Internet?
  - Audio?
  - CDROM?
  - From healthcare providers?

**Thank you**
